# Putative Role of Adenosine A1 Receptors in Exogenous Ketone Supplements-Evoked Anti-Epileptic Effect

**DOI:** 10.3390/ijms25189869

**Published:** 2024-09-12

**Authors:** Zsolt Kovács, Enikő Rauch, Dominic P. D’Agostino, Csilla Ari

**Affiliations:** 1 Department of Biology, BDTTC, ELTE Eötvös Loránd University, Károlyi Gáspár tér 4., 9700 Szombathely, Hungary or zskovacsneuro@gmail.com (Z.K.); raucheniko9810@gmail.com (E.R.); 2 Institute of Biology, University of Pécs, Ifjúság Str. 6, 7624 Pécs, Hungary; 3 Ketone Technologies LLC., Tampa, FL 33612, USA; ddagosti@usf.edu; 4 Department of Molecular Pharmacology and Physiology, Laboratory of Metabolic Medicine, Morsani College of Medicine, University of South Florida, Tampa, FL 33612, USA; 5 Institute for Human and Machine Cognition, Ocala, FL 34471, USA; 6 Department of Psychology, Behavioral Neuroscience Research Laboratory, University of South Florida, Tampa, FL 33620, USA

**Keywords:** exogenous ketone supplement, ketosis, A_1_R, epilepsy

## Abstract

Approximately 30% of patients with epilepsy are drug-refractory. There is an urgent need to elucidate the exact pathophysiology of different types of epilepsies and the mechanisms of action of both antiseizure medication and metabolic therapies to treat patients more effectively and safely. For example, it has been demonstrated that exogenous ketone supplement (EKS)-generated therapeutic ketosis, as a metabolic therapy, may decrease epileptic activity in both animal models and humans, but its exact mechanism of action is unknown. However, it was demonstrated that therapeutic ketosis, among others, can increase adenosine level, which may enhance activity of A1 adenosine receptors (A_1_Rs) in the brain. It has also been demonstrated previously that adenosine has anti-epileptic effect through A_1_Rs in different models of epilepsies. Thus, it is possible that (i) therapeutic ketosis generated by the administration of EKSs may exert its anti-epileptic effect through, among other mechanisms, increased adenosine level and A_1_R activity and that (ii) the enhanced activity of A_1_Rs may be a necessary anti-epileptic mechanism evoked by EKS administration-generated ketosis. Moreover, EKSs can evoke and maintain ketosis without severe side effects. These results also suggest that the therapeutic application of EKS-generated ketosis may be a promising opportunity to treat different types of epilepsies. In this literature review, we specifically focus on the putative role of A_1_Rs in the anti-epileptic effect of EKS-induced ketosis.

## 1. Introduction

Different types of central nervous system conditions, for example, traumatic brain injury, infections, and febrile seizure, may be able to evoke pathological processes leading to neurological diseases, such as epilepsy [1,2,3,4] and comorbid behavioral diseases (e.g., anxiety) [5]. The pathophysiology of epilepsies and mechanisms of action of different anti-epileptic drugs (AEDs) have been investigated extensively [2,4,6,7,8,9]. It has been demonstrated that several pathological processes leading to epileptic seizures can be inhibited or dampened by AEDs. However, one third of patients are drug refractory [6,7,10,11,12], due to, for example, multidrug resistance associated protein- and/or P-glycoprotein-generated effects [13,14,15]. Moreover, there are over 20 AEDs currently in use for epilepsy and all have a range of serious adverse effects [16,17,18,19]. Thus, to obtain more effective and safe therapeutic options for the 60–70 million people diagnosed with epilepsy worldwide [20,21], the development of new anti-epileptic strategies based on its well-explored mechanism(s) of action is needed.

It has been demonstrated that adenosine and its analogues can decrease epileptic activity, mainly through A1 adenosine receptors (A_1_Rs) [22,23,24,25,26]. Moreover, not only ketogenic diets, but exogenous ketone supplement (EKS)-generated ketosis can also increase adenosine level in the brain [27,28]. Consequently, ketosis can evoke an anti-epileptic effect, in part through A_1_Rs [29,30,31,32], which may be a primary therapeutic target in treating epilepsy effectively and safely by the administration of EKSs. For these reasons, in this literature review article we specifically focus on and summarize our knowledge on the putative influence of EKSs-generated ketosis on epileptic activity through A_1_Rs.

## 2. Anti-Epileptic Effects of A1Rs

It has been demonstrated that A_1_Rs are widely expressed in the central nervous system [33] and their distribution is uneven in different brain areas in animals, such as rodents, and in humans [34,35,36,37]. These results suggest that A_1_Rs have a role not only in physiological, but also pathophysiological processes in the brain, such as the modulation of sleep and epilepsy [26,38,39,40,41]. A_1_Rs are expressed on neurons both pre-synaptically and post-synaptically, as well as on glial cells. A_1_Rs are coupled to ‘inhibitory’ G-protein G_i_ and G_O_, leading to (i) the inhibition of adenylate cyclase activity, and as a result, a decrease in second messenger cyclic adenosine monophosphate (cAMP) level and protein kinase A (PKA) activity; (ii) the modulation of the activity of both K^+^ channels (activation) and voltage dependent Ca^2+^ channels (inhibition); and (iii) the stimulation of phospholipase C (PLC) activity resulting in enhanced levels of both second messenger diacylglycerol (DAG) and inositol 1,4,5-triphosphate (IP_3_) [36,42,43].

**Figure 1:**
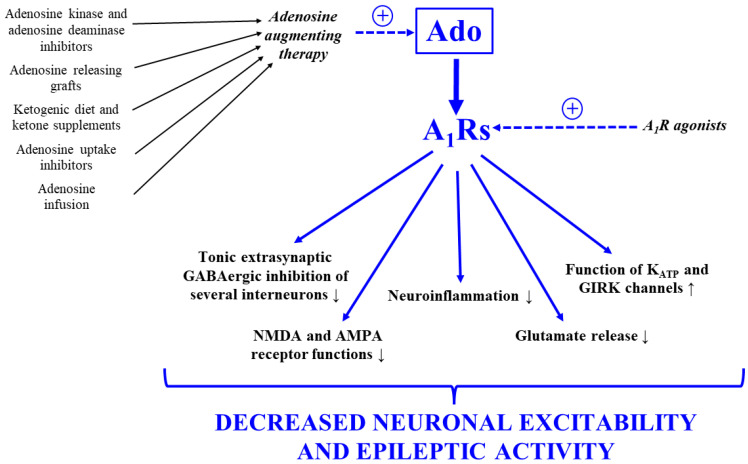
The main mechanisms of action of the A_1_R-induced anti-epileptic effect and activation of these processes by the administration of adenosine augmenting therapy and A1R agonists. Abbreviations: +, enhancement of adenosine level and A_1_R activity; A_1_R, A1 adenosine receptor; Ado, adenosine; AMPA, α-amino-3-hydroxy-5-methyl-4-isoxazolepropionic acid; GABA, gamma-aminobutyric acid; GIRK, G protein-coupled inwardly rectifying potassium; K_ATP_, ATP-sensitive potassium; NMDA, N-methyl-D-aspartate.

It is widely accepted that increased neuronal excitability in different brain areas, such as the hippocampus and several parts of the cerebral cortex (e.g., the somatosensory cortex), can lead to different types of epilepsies [6,7,10,11,44]. Endogenous adenosine, as an inhibitory neuromodulator molecule in the central nervous system, can evoke neuroprotective and anticonvulsant effects through A_1_Rs in different brain areas implicated in epilepsies [22,23,24,25,45]. Indeed, adenosine can attenuate seizure spreading, regulate seizure termination and reduce epileptic activity through A_1_Rs [25,46,47,48]. Adenosine levels increased during bicuculline-generated seizures in the hippocampi of human epileptic patients, as well as in rodent thalamocortical slices. This effect and the consequent increase in A_1_R activity may be a protective feedback mechanism to mitigate the duration and intensity of seizures [48,49,50,51,52]. The density of A_1_Rs were increased in the epileptic tissue in pentylenetetrazole, bicuculline, and kainic acid epilepsy rodent models, as well as in human temporal lobe epilepsy. It has been suggested that this endogenous adaptive mechanism may protect against subsequent seizures evoked by enhanced excitability [53,54,55,56]. Furthermore, the expression and activity of A_1_Rs was found to decrease before the appearance of absence epileptic activity in a well-established model of human absence epilepsy Wistar Albino Glaxo Rijswijk (WAG/Rij) rat [57]. It was demonstrated that A_1_Rs can impede neuronal postsynaptic N-methyl-D-aspartate (NMDA) and α-amino-3-hydroxy-5-methyl-4-isoxazolepropionic acid (AMPA) receptor functions [7,26,58]. Moreover, A_1_Rs can inhibit not only GABA(gamma-aminobutyric acid)ergic and glutamatergic signaling in the thalamus, but also thalamocortical inputs leading to anti-oscillatory and anti-epileptic effect [41,52,59,60]. In addition, A_1_Rs can enhance the function of different types of K^+^ channels post-synaptically, such as adenosine triphosphate (ATP)-sensitive potassium channels (K_ATP_) and G protein-coupled inwardly rectifying potassium (GIRK) channels. These changes may result in enhanced potassium efflux, hyperpolarization and stabilization of neuronal membrane potential providing protection against hyperexcitability in brain areas involved in epilepsy genesis [26,61,62,63,64,65,66] (Figure 1). A1Rs also suppress pre-synaptic excitatory neurotransmission (e.g., glutamate release) through the inhibition of voltage-gated Ca^2+^ channels (P/Q- and N-type) and transmitter release from synaptic vesicles, leading to decreased postsynaptic NMDA receptor activation and suppressed neuronal hyperexcitability in brain areas implicated in epilepsy genesis [26,40,67,68,69]. Furthermore, A1Rs to a lesser extent, can also decrease inhibitory GABAergic neurotransmission [26,70,71] in the brain. In relation to the last effect, a reduction in tonic extra-synaptic GABAergic inhibition of several GABAergic interneurons by A_1_Rs may evoke disinhibition of the involved interneurons. Consequently, the increased activity of these inhibitory GABAergic interneurons can enhance inhibition of pyramidal neurons and therefore decrease excitability, leading to diminished epileptic activity [26,71,72].

In relation to the anti-epileptic effect of A_1_Rs, it was also demonstrated that A_1_R knockout mice showed spontaneous seizures [30,73] and more severe consequences of kainic acid-induced status epilepticus, such as serious convulsions and death [74]. Antagonists of A_1_Rs, such as 8-cyclopentyl-1,3-dimethylxanthine (8-CPT), evoked proconvulsant influence in kindled rats [66,75], increased intensity of epileptiform activity and induced persistent epileptiform discharges in Mg^2+^-free conditions [76,77]. The administration of a non-selective adenosine receptor antagonist theophylline decreased, whereas a specific A_1_R antagonist 1,3-dipropyl-8-cyclopentylxanthine (DPCPX) increased, the number of spike-wave discharges (SWDs) dose-dependently in WAG/Rij rats [78,79]. Moreover, DPCPX induced seizure-like bursting activity in rat hippocampal slices [80] and generated proconvulsant effects in both audiogenic seizure- and pilocarpine-induced models in mice and rats, respectively [81,82]. The activation of A_1_Rs by adenosine and A_1_R agonists or the enhancement of adenosine level (adenosine augmenting therapy) in the brain—for example, by infusion of adenosine; inhibition of several adenosine metabolizing enzymes, such as adenosine kinase and adenosine deaminase; use of adenosine uptake inhibitors; adenosine releasing grafts; ketogenic diet; or EKSs—diminishes or prevents seizures [32,40,41,79,83,84] (Figure 1). For example, it was demonstrated that adenosine was able to generate an alleviating effect against pentylenetetrazole-, kainic acid-, bicuculline- and penicillin-evoked seizures in mice and/or rats [85,86,87,88,89]. The enhancement of A_1_R activity by the administration of A_1_R agonists, such as N^6^-cyclopentyl-adenosine (CPA), 2-chloro-N^6^-cyclopentyl-adenosine (CCPA), 2-chloroadenosine (2-CLA), D-N^6^-(2-phenylisopropyl) adenosine (D-PIA), L-N^6^-(2-phenylisopropyl) adenosine (L-PIA), R-N^6^-(2-phenylisopropyl) adenosine (R-PIA), and S-N^6^-(2-phenylisopropyl) adenosine (S-PIA), as well as N^6^-cyclohexyl-adenosine (CHA), was effective against different types of seizures. For example, the administration of CPA was effective in bicuculline-, kainic acid-, 4-aminopyridine-, pentylenetetrazole-, aminophylline- and 3-mercaptopropionic acid-induced seizures in rats and mice [86,90,91,92,93,94]. It has also been demonstrated that CCPA can evoke anti-seizure effects in bicuculline-, kainic acid-, pentylenetetrazole- and pilocarpine-induced seizures in rodents [23,70,95,96,97]. Moreover, 2-CLA and/or CHA as well as D-, L-, R- and S-PIA also suppressed pilocarpine- [82,98,99,100,101,102], pentylenetetrazole- [55,85,86,97,103], kainic-acid- [85], 3-mercaptopropionic acid- [85], 3-nitropropionic acid- [104], bicuculline- [90,105], penicillin- [106] and 4-aminopyridine [91]-induced seizures in different animal models. In addition, 2-CLA, CHA and L-PIA suppressed or prevented seizures in rat kindling models [107,108,109,110,111,112].

Furthermore, adenosine can be metabolized to adenosine monophosphate (AMP) or inosine by adenosine kinase and adenosine deaminase, respectively. Thus, the enhanced activation of both adenosine kinase and adenosine deaminase is able to decrease adenosine level effectively [113]. In contrast, the enhancement of adenosine level by the administration of adenosine kinase inhibitors, such as 5′-iodotubercidin and 5′-amino-5′-deoxyadenosine, or adenosine deaminase inhibitors, such as 1-[(2,6-difluorophenyl)-methyl]-1H-1,2,3-triazolo[4,5-c])pyridine-4-amine mono hydrochloride (BW534U87), 2′-deoxycoformycin and erythro-9-(2-hydroxy-3-nonyl)adenine (EHNA), impedes the metabolic clearance of adenosine and evokes the enhanced release of adenosine to the extracellular space [114,115,116]. Consequently, these inhibitors mitigated seizure activity in bicuculline- [117,118,119], and/or kainic acid- [24], pentylenetetrazole- [120] and Mg^2+^-free condition [116,121] -induced rodent epilepsy/seizure models, likely through A_1_Rs. Moreover, BW534U87 induced seizure reduction in a rat kindling model [120]. In relation to the link between increased adenosine kinase activity and enhanced epileptic activity, it has been demonstrated that the expression of adenosine kinase, expressed highly in astrocytes [122], was upregulated in chronic epilepsy mostly in astrocytes by astrogliosis [24,123,124,125]. These processes can result in a decrease in extracellular adenosine level and, consequently, chronic and recurrent seizures through diminished release of adenosine from astrocytes [126,127,128], leading to reduced A_1_R activity and pathological hyperexcitability. Thus, astrogliosis-evoked increase in adenosine kinase activity can generate neuronal dysfunction under epileptic conditions, suggesting that not only the effect of astrogliosis on neuronal activity, but also the accompanying epileptic seizures may be modulated and prevented through A_1_Rs [24,123,124]. It was also demonstrated that astrocytes may have a role in the generation, spreading, and maintenance of different types of epileptic seizures [129,130,131,132,133,134], suggesting that the modulation of activity and functions of astrocytes may allow us to decrease the occurrence of epileptic seizures, among other issues, through A_1_Rs. Indeed, epileptogenesis may reduce astrocytic A_1_R expression [135,136,137] and A_1_Rs can attenuate astrogliosis [138,139]. In addition, it was demonstrated that the activation of A_1_Rs can decrease neuroinflammation through the inhibition of not only astrocytic proliferation, but also microglial activation. Nevertheless, enhanced microglial activity and inflammatory processes were revealed in A_1_R knock-out mice [140,141,142,143]. Therefore, lipopolysaccharide (LPS) can increase the level of proinflammatory cytokines, such as interleukine-1β (IL-1β), leading to enhanced excitability and seizure activity, for example, by increasing the extracellular glutamate concentration [129,144,145,146]. The initiation of astrocytic IL-1β expression can also evoke or aggravate absence epileptic seizures [147]. It has also been demonstrated that both LPS and IL-1β may enhance the expression of adenosine kinase in astrocyte cell cultures [125]. This last result suggests that the adenosine kinase-generated decrease in both adenosine level and, therefore, the adenosine-generated inhibition through A_1_Rs may be one of the main mechanisms of action by which LPS and IL-1β can evoke increase in absence epileptic activity [40,147,148,149]. For example, the activation of A_1_Rs downregulated the genes in astrocytes involved in the LPS-generated inflammatory processes (e.g., inducible nitric-oxide synthase) [150] and alleviated the harmful effects of reactive oxygen species (ROS) on different brain cells [151]. However, it was suggested that astrogliosis, increased adenosine kinase activity and decreased adenosine level in the brain may be pathological hallmarks of epilepsy [152]. Furthermore, the inhibition of adenosine uptake by nucleoside transporter inhibitors, such as dipyridamole and S-(4- nitrobenzyl)-6-thioinosine (NBTI), can also increase extracellular adenosine level by which these drugs can generate alleviating influences in bicuculline, pentylenetetrazole, and kindling models [121,153,154,155].

Nevertheless, it was also demonstrated that the administration of effective doses of adenosine, adenosine uptake (transporter) inhibitors, adenosine metabolic enzyme inhibitors or A_1_R agonists not only evoke alleviating effects on epileptic activity, but can also induce adverse side effects or tolerance [24,43,85,123,156,157,158,159]. These limitations can restrict the therapeutic usability of these treatments and drugs. Furthermore, it has also been demonstrated that the basal and slightly increased extracellular adenosine level can preferentially enhance the activity of neuronal A_1_Rs, leading to inhibitory effects [67,160] and therefore decrease both neuronal excitability and epileptic activity through the different pathways described above (Figure 1). However, an excessive increase in adenosine level can lead to the activation of excitatory A_2A_ adenosine receptors (A_2A_Rs) in the A_1_-A_2A_ receptor heteromer, which may block A_1_R function and increase glutamate release [67,161,162]. These influences can increase both neuronal excitability and epileptic activity. Indeed, it has been demonstrated that increasing the activity of A_2A_Rs by the administration of an A_2A_R agonist (2-(4-(2-carboxyethyl)-phenylamino)-5′-N-ethylcarboxamido-adenosine (CGS 21680) evoked a proconvulsant effect in a rat kindling model [101,163] and enhanced absence epileptic activity in WAG/Rij rats [57]. Furthermore, an A_2A_R antagonist 5-amino-7-(2-phenylethyl)-2-(2-furyl)-pyrazolo-(4,3-c)1,2,4-triazolo(1,5-c)-pyrimidine (SCH 58261) reduced pilocarpine-induced seizures [102] and absence epileptic seizures in WAG/Rij rats [57]. Consequently, in relation to the safe administration of different types of adenosine-augmenting therapies against epilepsy, the adenosine level should be increased carefully to avoid the preferential activation of A_2A_Rs in the brain.

## 3. Exogenous Ketone Supplement-Evoked Ketosis Can Decrease Epileptic Activity through A1Rs

**Figure 2:**
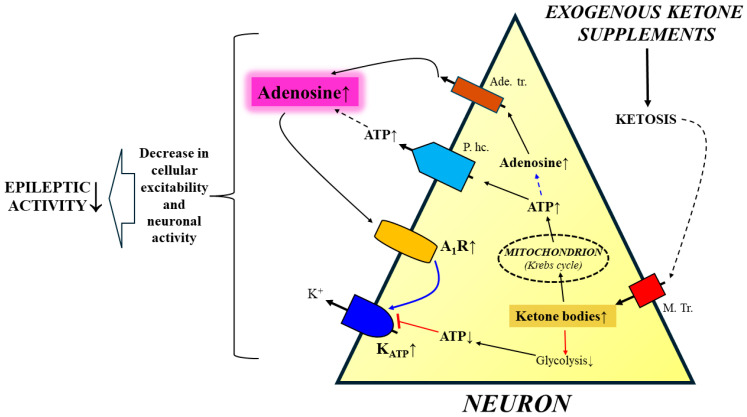
Putative main mechanisms of action of ketosis-evoked anti-epileptic effect through the increased adenosine level and A_1_R activity. Abbreviations: A_1_R, A1 adenosine receptor; Ade. tr., adenosine transporter; ATP, adenosine triphosphate; K_ATP_, ATP-sensitive potassium; M. Tr., monocarboxylate transporter; P. hc., pannexin hemichannel.

It has been demonstrated that ketogenic diets can mitigate seizure activity in mice by decreased adenosine kinase activity (a major drug target), leading to increased adenosine levels and A_1_R activity, as well as the opening of K_ATP_ channels [29,30,31,32]. It is possible that the last effects may be evoked by EKS administration-generated ketosis. Ingestion of EKSs, such as ketone esters (KEs) and ketone salts (KSs), delivers ketone bodies, such as beta-hydroxybutyrate (βHB) [164,165,166], generating rapid and sustained ketosis (1–7 mM; therapeutic ketosis) [167,168,169,170,171]. Ketone bodies may be transported to the bloodstream and subsequently to the brain and brain cells, through monocarboxylate transporters, where they can enhance mitochondrial ATP synthesis in neurons [172,173]. The ATP is released into the extracellular space by pannexin hemichannels [29,41], where it can be metabolized to adenosine by ecto-nucleotidases [27,28,174] leading to increased A_1_R activity. Consequently, EKS-evoked ketosis can decrease cellular excitability and neuronal activity, and therefore, epileptic activity in animal models and human patients [41,79,173,175,176,177,178,179], likely through all A_1_R-generated anti-epileptic effects as described above, such as the ATP/adenosine/A_1_R/K_ATP_ channel pathway (Figure 2) [27,28,31,180,181]. In addition, ATP can metabolize to adenosine intracellularly, and adenosine can be transported to the extracellular space through adenosine transporters [113], increasing adenosine level and A_1_R activity (Figure 2). Moreover, ketosis can inhibit glycolysis and, therefore, decrease ATP levels near the plasma membrane of neurons resulting in both increased activity of K_ATP_ channels and hyperpolarization of neuronal membranes, leading to decreased epileptic activity (Figure 2) [29,79,171,172,173,182,183]. Consequently, these results suggest that EKS-generated ketosis can mitigate epileptic activity in different types of epilepsies, in part through anti-glycolytic and ketosis-induced modification of A_1_Rs-evoked influences. In addition, it has been demonstrated that while EKSs containing βHB are more effective, the ketone precursor medium-chain triglyceride (MCT) supplementation is also able to mildly increase the level of ketone bodies in the blood [168,184]. It is likely that MCT oil may exert its anti-epileptic activity, at least partly, through A_1_Rs and administration of EKSs and MCT oil in combination may be effective in rapid and sustained elevation of ketosis [168] and, consequently, effectively decrease epileptic seizures. Moreover, combined administration of EKSs with MCT oil requires lower doses of both individual components, which may reduce potential side effects of these ingredients [168,177]. Indeed, it has been demonstrated that administration of EKSs, such as KEs (e.g., R,S-1,3-butanediol acetoacetate diester) and KSs (e.g., Na^+^/K^+^-D/L-βHB salt), as well as MCT oil alone or in combination can alleviate epileptic seizures. For example, KE (R,S-1,3-butanediol acetoacetate diester) alone; a mix of KS (Na^+^/K^+^-D/L-βHB salt) with MCT oil containing 60% caprylic triglyceride and 40% capric triglyceride in 1:1 ratio (named KSMCT: KS + MCT); and a KEKS (KE + KS)-containing standard rodent chow (20% KEKS) decreased absence epileptic activity (SWD number and total time of SWDs) in WAG/Rij rats [79,182,185] (Table 1).

It was also demonstrated that the administration of KE (R,S-1,3-butanediol acetoacetate diester) alone and in combination with MCT oil (named KEMCT: KE + MCT oil in 1:1 ratio) can increase the latency to hyperbaric oxygen-induced tonic–clonic seizures in rats [175,186]. Moreover, KE (R,S-1,3-butanediol acetoacetate diester) elevated the threshold of pentylenetetrazole-induced seizures in rats [187,188] and decreased audiogenic and kainate-induced seizure activity and severity in a mouse model [189]. Moreover, MCT oil alone may decrease seizure frequency (number of seizures/month) and the seizure day rate (number of seizure days/month) in dogs [190,191,192], as well as the daily seizure frequency in human patients (children and adults) with intractable epilepsy [193,194,195,196] (Table 1). In addition, it has been revealed that ketosis is able to reduce the occurrence of seizure-inducing neuroinflammatory processes [178,197,198,199,200,201]. It was also demonstrated that EKS-evoked ketosis can decrease not only spontaneously developed SWDs, but also LPS-evoked increases in SWD number in WAG/Rij rats, the effects of which were abolished by DPCPX administration [79,182,185]. These results also implicate A_1_Rs-evoked processes in the anti-epileptic influences of EKSs, such as KE-, KSMCT-, and KEKS-supplemented food (20% KEKS) [79,182,185]. It has been suggested that A_1_Rs could modulate the effects of EKSs and EKSs–MCT oil mixes on epileptic activity, anxiety (KE and KS alone, as well as KSMCT) [167,202] and other psychiatric diseases [203], as well as isoflurane-anesthesia-generated influences (KE and KS alone, as well as KEKS, KSMCT and KEMCT) [204,205,206]. These results also strengthened our hypothesis that not only ketogenic diet [29,207], but also EKSs can decrease epileptic activity through increased extracellular adenosine level and A_1_R activity in the brain. However, it is likely that the influence of EKS-generated ketosis through A_1_Rs may be one of numerous mechanisms responsible for ketosis-induced anti-epileptic activity. For example, βHB can bind to hydroxycarboxylic acid receptor 2 (HCAR2) and inhibit the NOD-like receptor pyrin domain 3 (NLRP3) inflammasome, resulting in suppressed proinflammatory mediators [198,200]. Moreover, βHB can inhibit class I histone deacetylases (HDACs) and promote histone and non-histone hyperacetylation. The overall result is suppressed inflammation while simultaneously increasing endogenous antioxidant protection [173,178,197,200]. Ketosis may inhibit excessive Ca^2+^ release and increase extracellular GABA levels [27,173,208,209] and MCT oil may exert its effect through the inhibition of AMPA receptors [210]. These reported effects may attenuate neuronal hyperexcitability together with A_1_R-generated effects. The A_1_R-induced alleviating influences may be directly and indirectly linked to the anti-epileptic effects of EKSs.

As described above, careful pharmacological modulation of the level of adenosine in the brain may be effective and safe against central nervous system diseases, such as epilepsy, without causing severe side effects. Ketosis appears to have a stabilizing effect on the modulation of brain adenosine level, but this may be dose-dependent. For example, it has been demonstrated that EKS-induced ketosis decreased the anxiety level [167], likely through A_1_Rs [202], but higher doses of EKS cannot enhance its alleviating effect on anxiety level, compared to lower doses in WAG/Rij rats [211]. These results suggest that a specific dose range of EKSs is needed to alleviate the symptoms of anxiety and, theoretically, other diseases. It is conceivable that higher doses of EKS could theoretically lead to “energy toxicity” and hypomania, particularly in individuals with a predisposition to mood disorders. Moreover, the level of EKS-evoked ketosis and, theoretically, the increase in adenosine level and A_1_R activity may be not only dose- but also sex- and age-dependent [212]. For example, the administration of the same dose of the KEMCT induced significantly higher blood R-βHB levels in female WAG/Rij rats, compared to male rats between the 2nd and 11th month of the experiment, but between 14th and 17th month lower R-βHB levels were detected in female rats than in male rats [212]. These results suggest the age- and sex-dependent beneficial effects of EKS-induced ketosis on central nervous system diseases, such as epilepsy, as well as on other pathophysiological and physiological processes, likely through A_1_Rs. In addition, the administration of EKSs does not induce strong adenosinergic peripheral or central nervous system side effects in animal models or human patients (e.g., side effects are mainly mild gastrointestinal problems) [165,166,169,170,213]. Nevertheless, the ketogenic diet can cause more severe adverse effects, such as the alteration of menstruation, growth retardation, weight loss, nausea, gastritis, constipation, nephrolithiasis, hyperlipidemia, hypoglycemia, and hyperuricemia [214,215,216]. Of potential relevance is the observation that both the ketogenic diet and ketone esters are able to decrease not only seizure activity, but also the plasma levels of the “hunger hormone” and anti-seizure molecule ghrelin [217,218,219]. A decreased level of ghrelin can suppress appetite and, theoretically, could mitigate the anti-seizure effect of ketogenic diets and ketone esters [217,218,219]. Thus, it may be helpful to find EKS formulations that do not reduce ghrelin levels. Furthermore, regarding the ketogenic diet, the need for highly motivated patients and extensive medical guidance often limits the long-term compliance needed to achieve a therapeutic effect. Therapeutic ketosis can be induced with less difficulty and greater precision with the dose-dependent administration of EKSs [167,175,182,220], and this can also be conducted to further augment dietary therapy, even for patients with neural injuries [221]. All these results above suggest that the putative modulation of A_1_R activity by the administration of EKSs may be a more promising and effective, as well as safer, alternative metabolic therapy to mitigate epileptic activity compared to the classic ketogenic diet [193,222]. Nevertheless, long-term animal and clinical studies are needed to assess the dose-dependent effects of EKSs (alone and in combination) for different seizure disorders to understand seizure-specific efficacy, dose parameters, and safety.

## 4. Conclusions

The influence of EKS-generated ketosis on epileptic activity and its mechanism(s) of action has not been fully investigated. However, in relation to the effect of EKS-generated ketosis on epileptic activity through A_1_Rs, it was demonstrated unambiguously that (i) ketosis can increase adenosine level in the brain, (ii) increased adenosine level can enhance A_1_R activity, and (iii) that adenosine has an anti-epileptic effect through different A_1_R-evoked pathways. Consequently, we can conclude that (i) the A_1_R-generated alleviating effect via EKS-evoked ketosis reduces epileptic activity and (ii) increasing adenosine levels by EKS -induced therapeutic ketosis, as a new type of adenosine augmentation therapy, may be a promising additional therapeutic tool to treat epilepsy. EKSs can modulate endogenous processes through the metabolic modulation of A_1_Rs, suggesting that EKS-evoked ketosis may be an effective and safe therapeutic method for seizure suppression through the adenosine/A_1_R/K_ATP_ channel pathway. However, new and detailed studies are needed to strengthen the role for EKS-induced modulation of the A_1_R-generated anti-seizure effects in different disorders. For example, the effects of the formulation, dose, timing, and mode of administration of EKSs on the adenosine level should be measured in both sexes and at different ages in different brain areas implicated in epilepsy. In addition, the accurate investigation of EKS-generated changes in signaling processes associated with ketosis-evoked anti-epileptic effects through A_1_Rs can further support the modulation of A_1_R activity as a promising therapeutic target for the treatment of epilepsy, especially in drug-refractory patients. Based on the current literature, it is conceivable that EKSs could be used as a monotherapy for those that have experienced side effects with AEDs, or as a means to further augment the therapeutic efficacy of ketogenic diet therapy. However, new animal and clinical studies are needed to demonstrate and strengthen the usability of EKSs against different types of epilepsies.

## Figures and Tables

**Table 1:** Results of some preclinical and human studies evaluating the anti-epileptic effect of KE, KEMCT, KEKS and MCT oil.

Ketogenic Agents	Species (Model System, Seizure Type)	Results	References
KE, KSMCT, KEKS	Rat (genetic model of human absence epilepsy: WAG/Rij rat; absence seizures)	SWD number and total time of SWDs ↓	[79,182,185]
KE, KEMCT	Rat (hyperbaric hyperoxia-induced seizures)	Latency to seizures ↑	[175,186]
KE	Rat (pentylenetetrazole-induced seizures)	Threshold of pentylenetetrazole-induced seizures ↑	[187,188]
	Mouse (wild-type and Angelman syndrome mouse; audiogenic- and kainic acid-induced seizures)	Latency to seizures ↑ Seizure activity and severity ↓	[189]
MCT oil	Dog (focal and generalized tonic-clonic seizures)	Seizure frequency (number of seizures/month) ↓ Seizure day rate (number of seizure days/month) ↓	[190,191,192]
	Human (drug-resistant focal and generalized tonic-clonic seizures)	Daily seizure frequency ↓	[193,194,195,196]

Abbreviations: KE, ketone ester, R,S-1,3-butanediol acetoacetate diester; KEKS, 10% ketone ester (KE, R,S-1,3-butanediol acetoacetate diester) and 10% ketone salt (KS, Na^+^/K^+^-βHB salt) in standard rodent chow (KEKS supplemented food); KEMCT, mix of KE (R,S-1,3-butanediol acetoacetate diester) and MCT (medium-chain triglyceride) oil in 1:1 ratio; MCT, medium-chain triglyceride; SWD, spike-wave discharge; WAG/Rij, Wistar Albino Glaxo Rijswijk.

## Abbreviations

2-CLA: 2-chloroadenosine; A1R, A1 adenosine receptor; A2AR, A2A adenosine receptors; AMPA, α-amino-3-hydroxy-5-methyl-4-isoxazolepropionic acid; ATP, adenosine triphosphate; βHB, beta-hydroxybutyrate; BW534U87, 1-[(2,6-difluorophenyl)-methyl]-1H-1,2,3-triazolo[4,5-c])pyridine-4-amine mono hydrochloride; CCPA, 2-chloro-N^6^-cyclopentyl-adenosine; CHA, N^6^-cyclohexyl-adenosine; CPA, N^6^-cyclopentyl-adenosine; DPCPX, 1,3-dipropyl-8-cyclopentylxanthine; D-PIA, D-N^6^-(2-phenylisopropyl) adenosine; EKS, exogenous ketone supplement; GABA, gamma-aminobutyric acid; IL-1β, interleukine-1β; KATP channel, ATP-sensitive potassium channel; KE, ketone ester; KEKS, ketone ester/KE + ketone salt/KS; KEMCT, ketone ester/KE + medium-chain triglyceride/MCT oil; KS, ketone salt; KSMCT, ketone ester/KE + medium-chain triglyceride/MCT oil; L-PIA, L-N^6^-(2-phenylisopropyl) adenosine; LPS, lipopolysaccharide; MCT, medium-chain triglyceride; NMDA, N-methyl-D-aspartate; R-PIA, R-N^6^-(2-phenylisopropyl) adenosine; S-PIA, S-N^6^-(2-phenylisopropyl) adenosine; SWD, spike-wave discharge; WAG/Rij, Wistar Albino Glaxo Rijswijk.
